# Spirometry has added value over electrodermal activity as a physiological marker of mental load in male subjects

**DOI:** 10.1038/s41598-022-08480-x

**Published:** 2022-03-16

**Authors:** Tobias Neukirchen, Moritz Stork, Matthias W. Hoppe, Christian Vorstius

**Affiliations:** 1 Department of General and Biological Psychology, University of Wuppertal, Max-Horkheimer-Str. 20, 42119 Wuppertal, Germany; 2 Institute of Movement and Training Science, University of Leipzig, Leipzig, Germany

**Keywords:** Respiration, Human behaviour, Diagnostic markers

## Abstract

The objective distinction of different types of mental demands as well as their intensity is relevant for research and practical application but poses a challenge for established physiological methods. We investigated whether respiratory gases (oxygen uptake and carbon dioxide output) are suitable to distinguish between emotional stress and cognitive load. To this end, we compared the application of spirometry with an established procedure, namely electrodermal activity (EDA). Our results indicate that electrodermal activity shows a strong responsivity to emotional stress induction, which was highly correlated with its responsivity to cognitive load. Respiratory gases were both sensitive and specific to cognitive load and had the advantage of being predictive for cognitive performance as well as self-reported emotional state. These results support the notion that respiratory gases are a valuable complement to common physiological procedures in the detection and discrimination of different mental demands.

## Introduction

### Background

Research on using spirometry and corresponding respiratory gases, such as oxygen uptake (VO_2_) and carbon dioxide output (VCO_2_), for measuring psychological parameters is limited. In contrast, measuring local metabolic activity and using self-report to learn about latent cognitive and emotional processes are common methods in psychological research. Imaging methods for metabolic processes within the brain (e.g., fMRI, NIRS, fPET) are established to investigate cognitive processes, whereas peripheral physiological measurement methods (e.g., measurement of skin conductivity and pulse rate) are commonplace in studying emotional processes^1^.

Despite their established application in other scientific disciplines such as sport science and medicine^2^, respiratory gas measures are sparsely used in psychological research. This fact could be highly disproportionate to its potential use, as indicated by a recent review^3^. Yet, as Suess and colleagues^4^ pointed out over four decades ago, respiration rate alone seems to be an insufficient measure of respiratory reactivity to psychological stimuli and more sophisticated parameters are needed. Such parameters based on respiratory gases, particularly VO_2_ and VCO_2_, seem to be more sensitive to cognitive load and might even allow conclusions about task-related physiological and psychological processes^3^, making them promising candidates for the investigation of metabolic demands in cognitive task processing.

As psychological influences on respiration are far better understood in relation to emotional reactions, it might seem counterintuitive to utilize them in the context of cognitive load. Especially, as it is well established that emotional processes can alter respiratory parameters such as depth and frequency^5,6^, which in turn might impact VO_2_ and VCO_2_^2^. From an evolutionary point of view, this supports the idea that emotions serve as means to enhance physical preparedness, e.g., to provide additional oxygen to fuel an imminent fight or flight response^7^. In this case, the skeletal muscles can metabolize the additional oxygen as they work. In a similarly vein, the brain metabolizes oxygen to energize cognitive processing. In both cases, the metabolite carbon dioxide is produced^8^.

This raises the question, how emotional and task-related cognitive processing work can be measured and distinguished, when analyzing respiratory parameters that are influenced by both processes? Grasmann and colleagues^3^ suggested that a crucial distinction can be made, whether a respiratory response is *adaptive or maladaptive*. This is because an increased effort to upregulate VO_2_ (e.g., by changing breathing patterns) is maladaptive if it is triggered in preparation for a redundant (emotion mediated) fight or flight response. An example of this is the induction of emotional stress in a physically resting participant. In this maladaptive case, the VCO_2_ of exhaled gases is diminished, as metabolic processes are outpaced by respiration. An adaptively increased VO_2_, as with increased cognitive effort in a physical resting participant, however, should show a matching rate of VCO_2_. In other words, when using spirometry to measure cognitive load, it should be possible to detect emotion induced changes in respiration by observing an increased VO_2_ without a matching increase in VCO_2_, because emotional stress alone does not seem to change metabolic demand and increase O_2_ consumption^9^.

Hence, in line with Grasmann and colleagues^3^, we promote the idea that it should be possible to distinguish between cognitive load and mental stress using spirometry. Furthermore, depending on the magnitude of the suggested effects, respiratory gas parameters could even provide insight into the effort exerted for a cognitive task. This is also in line with our work on cognitive glucose sensitivity, in which we demonstrated profound inter-individual differences in the effects of carbohydrate ingestion and cognitive performance^10^. It is reasonable to expect that VCO_2_ corresponds to effort-related increases in glucose metabolism^11^.

Additionally, we are pioneering the combination of spirometry and classical peripheral psychophysiological measures in the context of cognitive and emotional processing. Therefore, we investigated whether VO_2_, VCO_2_ and their responsiveness are a suitable tool to enhance detection and better distinguish between periods of emotional stress and cognitive task related processing compared to EDA measures.

### Hypotheses

It was not clear, whether an absolute difference between conditions was detectable (absolute perspective), or if the individual change from baseline to a specific condition had to be considered (relative perspective) as a better parameter. Therefore, we explored both (simple mean comparison and comparison of the individual change to baseline) in the hypotheses for our three research questions:

Firstly, we investigated whether respiratory gas parameters are sensitive to a change of demand condition on an individual level (baseline, cognitive load, emotional stress). This should manifest in an task-dependent individual change in respiratory gases relative to the individual baseline (hypothesis 1a). In addition to this relative statement, an absolute aspect was added, namely, to check whether specific load conditions can be reliably assigned to certain respiratory values on an inter-individual bases (hypothesis 1b).

Secondly, we examined whether spirometry can differentiate between cognitive load and emotional stress, testing the hypothesis that Corsi-Block-Tapping-Task (CBT) and the Threat-of-Shock paradigm (ToS) can be distinguished based on VCO_2_ rather than VO_2_^3^ and comparing it to the established measure of electrodermal activity (hypothesis 2a).

Complementary, we explored the idea of differential adaptivity of a respiratory response to psychological stimuli in more depth by testing whether the use of Respiratory Exchange Ratio(RER), as an individual index of the ratio between VCO_2_ production and VO_2_ consumption, can offer added diagnostic value (hypothesis 2b).

Finally, we investigated the external and discriminant validity of the spirometry in this unconventional application (hypothesis 3a and b). For the former, we tested whether variance in cognitive performance outcomes corresponds with VO_2_ and VCO_2_. Additionally, the external validity to detect self-reported levels of emotional stress of all physiological variables was tested (hypothesis 3a). For the discriminant validity, interrelations between parameter responsiveness to both cognitive load and emotional stress were examined (hypothesis 3b).

## Method

All methods were approved by the universities internal review board (MS/BBL 191119) and in accordance with the current version of the Declaration of Helsinki. Furthermore, informed consent was obtained from all participants at the beginning of the experimental session.

### Study design

In a within-subject design, all participants successively went through relaxation, baseline and two types of experimental conditions (cognitive load, emotional stress). The whole experimental procedure was divided into a total of six episodes (e.g., relax, baseline, relax, emotional stress, relax, cognitive load). Cognitive load and emotional stress were induced in counterbalanced order across participants.

### Sample

Due to gender effects and possible hormonal, respiratory, and metabolic changes during the menstrual cycle^12–14^, only male participants were recruited. The original sample consisted of 34 healthy participants with an average age of 26.35 (*SD* = 8.75). Mean height was 180.85 cm (*SD* = 8.99) with a mean weight of 80.55 kg (*SD* = 11.56). Of the participants, 90.9% were right-handed and 78.8% were non-smokers. All participants with a cognitive performance value of zero had to be excluded from analysis, as their immediate failure in the first trial of the cognitive task did not allow for the collection of useful data for EDA and spirometry. In addition, it can be assumed that they did not understand or follow task instructions. Due to technical problems with EDA measurement, 2 participants had to be excluded, resulting in a final sample size of *N* = 25 for analyzes involving EDA and *N* = 27 for all others.

### Data preparation and statistical analysis

Part of the first hypothesis, concerning the relative perspective, referred to whether the change in respiratory parameters differs between going from baseline to a cognitive task versus going from baseline to emotional stress induction. The respective change of going from baseline to either condition was expressed in percent of baseline values and we refer to this variable as the *responsiveness* of the parameter to a certain condition.

Expanding on the adaptivity hypothesis of Grasmann and colleagues^3^ we used the RER, computed as quotient of VCO_2_ and VO_2_ for our hypothesis 2b. The expression of this quotient should be indicative of the adaptivity of the respiratory response.

All analyzes were conducted using R^15^ and figures were produced using the package ggplot2^16^. For all reported correlations, Pearson's correlation coefficient was used. Mean comparisons were carried out using repeated measures t-test. The significance level for all tests was set at α = 0.05. Although normal distribution was violated for some variables, we still report t-test results as the amount of data points can be regarded as sufficient to be robust against violations of normality. In addition, when testing with non-parametric tests, the result pattern remained the same. As multiple hypotheses were tested, the false discovery rate (FDR) was determined^17^.

### Procedure

To minimize confounding variables, participants agreed in advance to consume only water 2 h, no drugs (including alcohol, caffeine, and nicotine) 12 h before the study, and to refrain from exercise for 24 h prior to participation. On arrival, demographic data were collected, and body weight and height were measured. Participants were guided to an air-conditioned room with a temperature set to 22° Celsius. Next, equipment for physiological measurements (EDA, spirometry) was attached to the participants and calibrated according to the manufacturers. All physiological measurements were taken simultaneously. Afterwards, participants were seated comfortably facing a computer screen (14-inch, resolution 1920 × 1080, 60 Hz) and the automated experimental protocol (Inquisit 5, Millisecond Software, Seattle, USA) started with a 4.5 min period for EDA electrode stabilization before the recording. A trackpad was used as input device to minimize participant movement during the experiment.

A relaxation episode, consisting of a slide show of neutral landscape images, constituted the beginning of the experimental protocol. A relaxation episode was included after each condition for 90 s. Baseline measurements were obtained during a minimally demanding vigilance task to prevent potential activation due to excitement or anticipation of the demands^18^. Next, again separated by an relaxation episode, cognitive load (Corsi-Block-Tapping-Task)^19^ and emotional stress (Threat-of-Shock Paradigm)^4^ were induced. Finally, measures of subjective fear of shock, estimation of shock probability, and well-being were obtained using questionnaires. Emotional stress induction and baseline episode had a duration of 3 min. The duration (minutes) of the cognitive load episode varied with individual performance (*M* = 2.57, *SD* = 1.30). All other episodes lasted for 3 min and the first and last 5 s of each episode were excluded from analyzes to account for artifacts and the delay between local metabolic activity and changes in respiratory gas parameters.

Participants were informed about the background and the subject of the study after the survey was conducted. However, former participants were instructed not to talk to any other potential participants about the test procedure, as the method of emotional stress induction (see below) relies on the illusive anticipation of electric shocks. Participation was voluntary and students could receive partial course credit. All procedures were approved by the university’s Ethics Committee.

### Physiological measurements

A Mindfield eSense Skin Response Sensor (Mindfield Biosystems, Gronau, Germany) was used to measure EDA. Dry electrodes, placed on volar surfaces of distal phalanges of index and middle finger of the non-dominant hand were attached using velcro strips. The portable sensor was connected to a computer via the headphone jack. EDA data were collected with DC and recorded with 5 Hz. Skin conductance level (SCL) was calculated as the mean skin conductance value for each participant in each specified task. For additional analyzes, parameters distinguishing between phasic (p_mean) and tonic (t_mean) portions of the EDA-signal were calculated following the algorithm suggested by Greco and colleagues^20^.

For the analysis of respiratory gases, we used a PowerCube Ergo respiratory gas analyzer (Ganshorn, Niederlauer, Germany). VO_2_ and VCO_2_ were measured by breath-by-breath technology and averaged over 10 s. The gas analyzing system was calibrated with a calibration gas (15.5% O_2_, 5% CO_2_ in N; Messner, Switzerland) and a precision 1-L syringe (Ganshorn, Germany) before each test. Data from the gas analyzer were processed using LF8 software (Ganshorn, Niederlauer, Germany).

### Emotional stress induction

Using the Threat-of-Shock paradigm (ToS), the expectation of electric shocks was intended to induce emotional stress in the form of anxiety. We used a self-report item to obtain a measure of fear of anticipated shock (4-point scale). The ToS setup was consistent with that of Suess et al.^4^.

### Cognitive load

The Corsi-Block-Tapping-Task (CBT) served to induce progressive cognitive load. It serves as a measure of visuo-spatial short-term memory performance^21^. We used a computerized implementation of the task as described by Kessels, van Zandvoort, Postma, Kappelle, & de Haan^19^. Participants were instructed to correctly reproduce a sequence of highlighted blocks using a touch pad. Sequence length (difficulty) increased by one with every successful trial, up to a sequence of nine blocks. Parallel versions contained different random lighting sequences. Total score, computed from the longest, correctly reproduced span and the total errors made, served as index for proficiency.

## Results

Descriptive results for the psychophysiological variables that were subject to subsequent hypothesis tests are summarized in Table 1. An overview of the analyses presented in the following section and their associated hypotheses can be found in Table 2. All mean comparisons refer to the averaged physiological values for the duration of the respective experimental condition.

**Table 1:** Descriptive statistics of the psychophysiological measurements across experimental conditions.

	Initial Relax			Baseline			CBT			ToS		
	*M*	*SD*	Range	*M*	*SD*	Range	*M*	*SD*	Range	*M*	*SD*	Range
VO_2_	0.31	0.05	0.20	0.27	0.05	0.21	0.33	0.06	0.26	0.30	0.07	0.25
VCO_2_	0.30	0.06	0.23	0.27	0.06	0.24	0.30	0.06	0.27	0.28	0.07	0.27
SCL	5.26	2.70	11.97	6.51	2.97	13.33	6.55	2.98	13.58	7.29	3.14	14.05
RER	0.93	0.07	0.26	0.98	0.11	0.54	0.92	0.11	0.53	0.92	0.09	0.40
RR	12.90	3.22	13.06	13.22	3.04	13.50	16.24	3.67	13.97	13.70	3.23	15.47
MV	8.59	2.02	8.69	9.25	2.28	9.42	10.03	2.07	7.91	9.22	2.41	8.53

SCL = Skin conductance level (µS), VO_2_ = Volume of oxygen uptake (l/min), VCO_2_ = Volume of carbon dioxide output (l/min), RER = Respiratory Exchange Ratio (VCO_2_/VO_2_), RR = Respiratory Rate, MV = Minute Volume, CBT = Corsi Block-Tapping Task, ToS = Thread of Shock, N = 27 for every condition except EDA measures (N = 25).

**Table 2:** Inference statistical results sorted by hypotheses and relevant physiological variables/conditions.

Hypothesis	Variable	Condition	p-value	FDR
Task-dependent-difference to baseline—Relative Perspective: Mean difference between responsiveness to experimental conditions	relative increase in VO_2_	cognitive load - emotional stress	** < .001**	**.018**
	relative increase in VCO_2_	cognitive load - emotional stress	.104	.798
	relative increase EDA	cognitive load - emotional stress	** < .001**	**.019**
Task-dependent-difference to baseline—Absolute Perspective: Mean difference between experimental condition and baseline	VO_2_	cognitive load - baseline	**.013**	.303
	VO_2_	emotional stress - baseline	**.007**	.109
	VCO_2_	cognitive load - baseline	**.019**	.271
	VCO_2_	emotional stress - baseline	.388	1.000
	SCL	cognitive load - baseline	.772	1.000
	SCL	emotional stress - baseline	**.003**	.061
Difference between Cognitive Load and Emotional Stress Mean difference between experimental conditions	VO_2_	cognitive load - emotional stress	**.006**	.105
	VCO_2_	cognitive load - emotional stress	.100	.798
	SCL	cognitive load - emotional stress	** < .001**	**.007**
Adaptivity of Respiratory Response: Comparing quotient of gases between conditions	RER	cognitive load - emotional stress	.775	1.00
	RER	cognitive load - baseline	**.022**	.280
	RER	emotional stress - baseline	**.007**	.111
External Validity				
Cognitive Performance	VO_2_—cognitive task performance	cognitive load	**.023**	.280
	VCO_2_—cognitive task performance	cognitive load	**.037**	.369
	SCL—cognitive task performance	cognitive load	.292	1.000
Fear of Shock	VO_2_—fear of shock	emotional stress	**.022**	.280
	VCO_2_—fear of shock	emotional stress	**.003**	.059
	SCL—fear of shock	emotional stress	.317	1.000
Discriminant validity: Intercorrelation between responsivenesses in experimental conditions (within same parameter)	relative increase in VO_2_	cognitive load - emotional stress	.456	1.000
	relative increase in VCO_2_	cognitive load - emotional stress	**.048**	.434
	relative increase in SCL	cognitive load - emotional stress	** < .001**	**.002**

Experimental conditions refer to emotional stress induction and cognitive load and are to be differentiated from baseline condition. We defined responsiveness of a physiological parameter to an experimental condition as the difference between its mean values in baseline and the corresponding experimental condition, expressed in percent of its baseline value. False discovery rate (FDR) is given for each tested hypothesis. Sample size for all tests was *N* = 27 except for EDA (*N* = 25). SCL: mean skin conductance level (µS), VO2: Volume of oxygen uptake (l/min), VCO2: Volume of carbon dioxide output (l/min), RER: Respiratory Exchange Ratio (VCO2/VO2).

**Figure 1:**
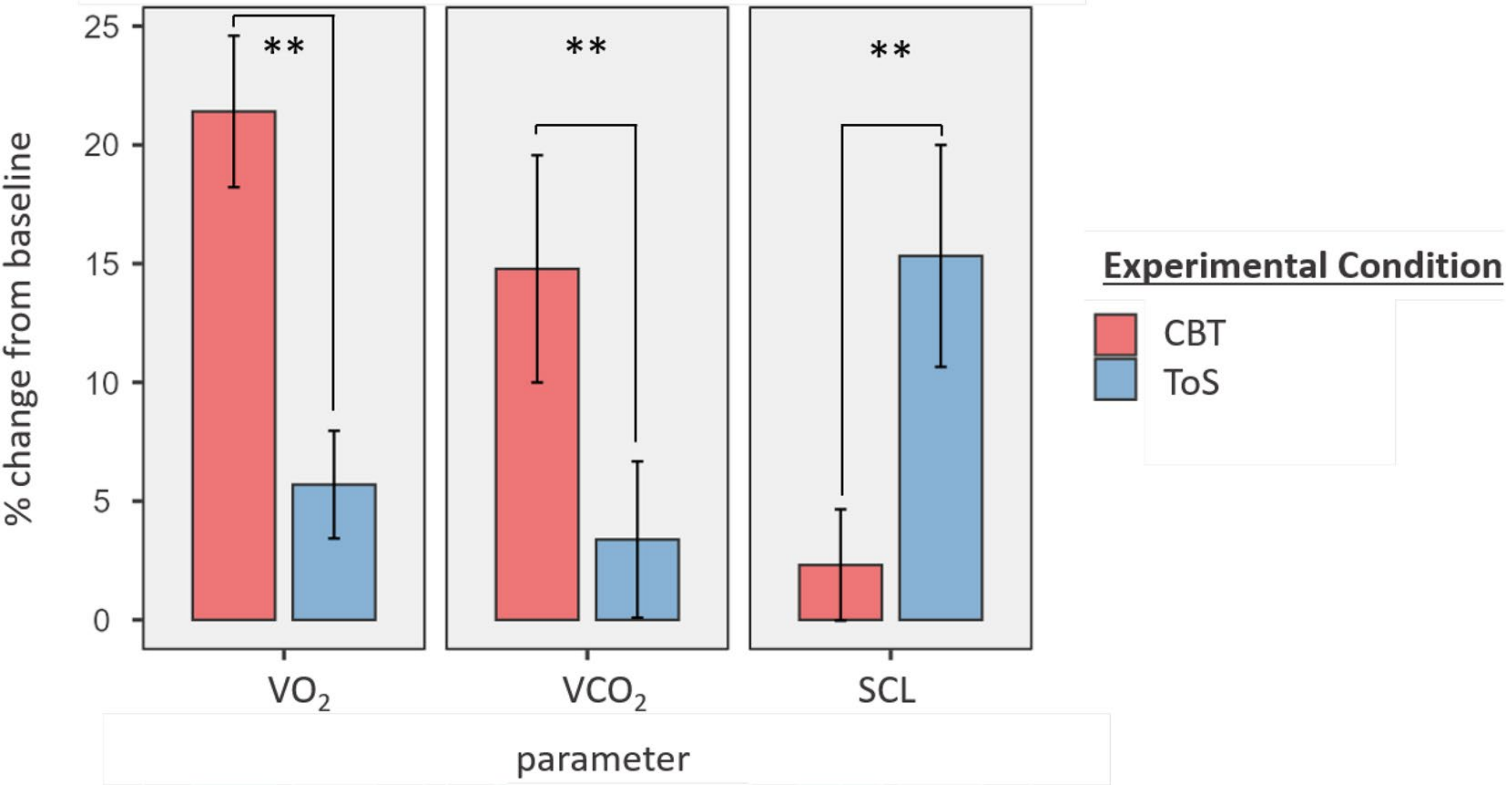
Responsiveness of physiological parameters (relative perspective). *Note.* Responsiveness corresponds to the parameter value change in percent when shifting from the baseline to the respective experimental condition. Except for VCO_2_, all mean comparisons between experimental conditions were significant. CBT = Corsi-Block-Tapping-Task; ToS = Threat-of-Shock; error bars indicate standard deviation.

**Figure 2:**
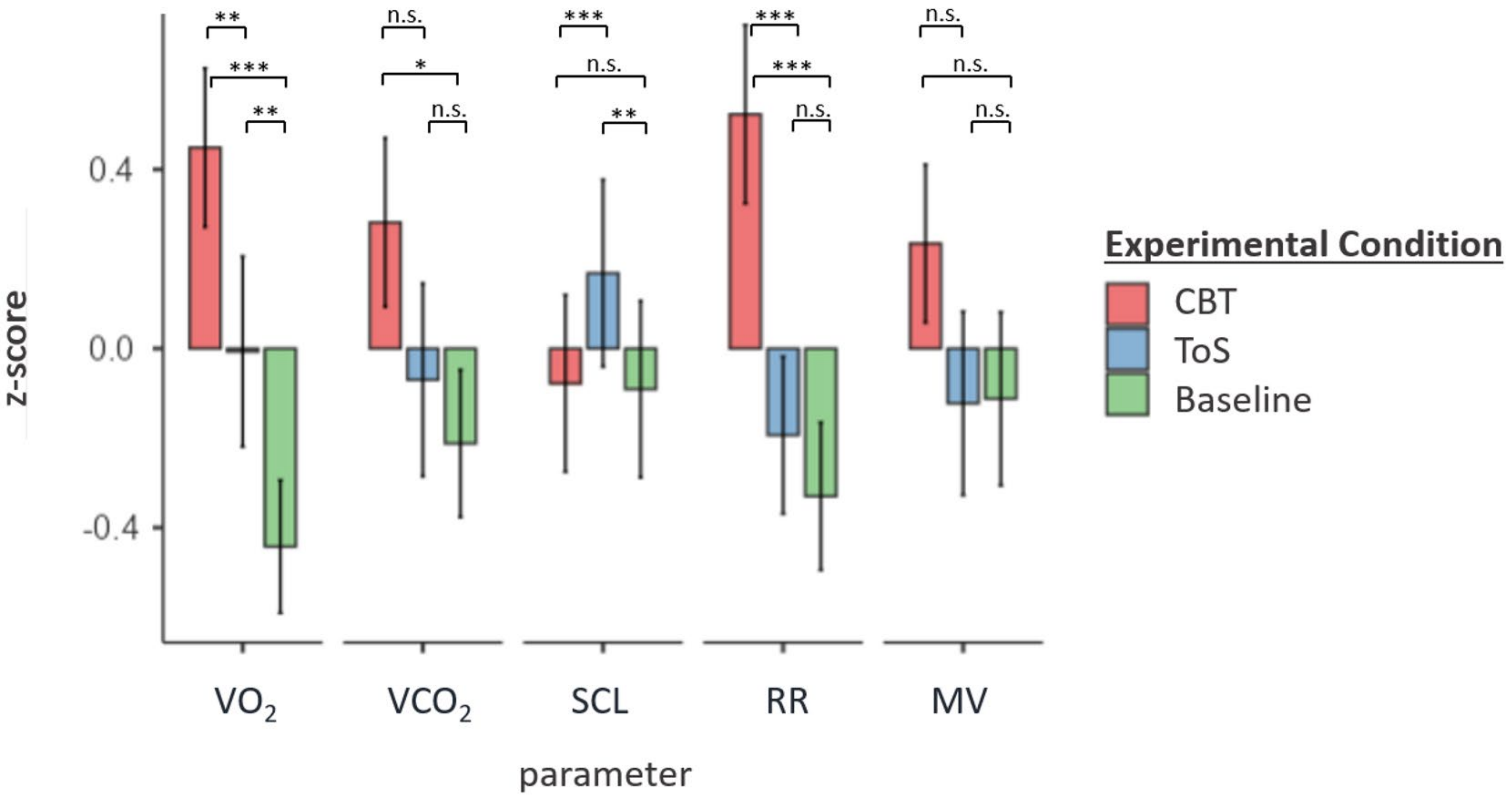
Mean values of physiological measures across experimental conditions (absolute perspective). *Note.* Due to scaling differences of EDA and gas parameters, mean values presented in this figure were z-standardized for each parameter. CBT = Corsi-Block-Tapping-Task, ToS = Threat-of-Shock; VO_2_ = Volume of oxygen uptake, VCO_2_ = Volume of carbon dioxide output, SCL = Skin conductance level, RR = Respiratory Rate, MV = Minute Volume; error bars indicate standard deviation. *p < .05, **p < .01, ***p < .001.

First, we tested the hypothesis that respiratory gases are sensitive to a change in demand condition on an individual level (baseline, CBT, ToS) from both, a relative and an absolute perspective (Figs. 1 and 2).

Regarding the relative perspective (hypothesis 1a), paired samples t-tests indicated significant differences for the responsiveness of VO_2_ with the CBT (*M*_*O*_ = 21.40%) inducing a greater increase than the ToS (*M*_*O*_ = 5.69%, *t*(26) = 3.76, *p* < 0.001), both relative to baseline.

Results for the responsiveness in VCO_2_ again showed a higher increase between baseline and CBT (*M*_*C*_ = 14.78%) than for baseline and ToS (*M*_*C*_ = 3.38%) although it failed to reach statistical significance (*t*(26) = 1.68, *p* = 0.104).

In contrast, SCL results showed a greater responsiveness to ToS (*M* = 15.32%) than to CBT (*M* = 2.31%), again in reference to the baseline (*t*(24) = −3.76, *p* < 0.001).

Next, hypothesis 1b tested the absolute perspective, comparing the mean respiratory gases of each testing condition (CBT, ToS) to baseline. Mean VO_2_ during CBT (*M*_*O*_ = 0.33, *t*(26) = −7.18, *p* < 0.001 ) and ToS (*M*_*O*_ = 0.30, *t*(26) = −2.94, *p* = 0.007) differed significantly from baseline values (*M*_*O*_ = 0.27). For the mean VCO_2_, only the difference between baseline (*M*_*C*_ = 0.27) and CBT condition reached significance (*M*_*C*_ = 0.30, *t*(26) = −2.49, *p* = 0.019).

For SCL, there was no significant mean difference between baseline and CBT (*t*(24) = −0.29 *p* = 0.772), whereas mean SCL during ToS (*M* = 7.29) differed significantly from both baseline (*M* = 6.51, *t*(24) = −3.25, *p* = 0.003) and CBT (*M* = 6.55, *t*(24) = −3.94, *p* < 0.001).

Additional analyzes using paired sample t-tests revealed significant differences for respiratory rate between baseline (*M* = 13.22) and CBT (*M* = 16.24, *t*(24) = −7.31, *p* < 0.001), as well as between ToS (*M* = 13.70) and CBT, *(t*(24) = 5.59, *p* < 0.001) but not for the comparison baseline vs. ToS.

There were no significant differences for minute volume across experimental conditions.

The assumption that emotional and cognitive load can be distinguished based on VCO_2_ rather than VO_2_ (hypothesis 2a) was tested using paired samples t-tests. The difference between CBT and ToS reached significance for the VO_2_ (*t*(26) = −2.69, *p* = 0.006) but fell short to do so for VCO_2_ (*p* = 0.100). The difference between mean SCL during CBT and ToS was significant (*t*(24) = −3.93, *p* < 0.001).

Complementary, we explored whether there is evidence for the hypothetical maladaptive respiratory response to emotional stress using the quotient of VCO_2_ and VO_2_—the RER (hypothesis 2b). Paired samples t-test indicated significant differences in the RER between baseline (*M* = 0.98) and CBT (*M* = 0.92, *t*(26) = 2.45, *p* = 0.022) and between baseline and ToS (*M* = 0.92, *t*(26) = −2.91, *p* = 0.007). However, no significant differences between the RER of ToS and CBT were found (*p* = 0.775).

Regarding hypthesis 3a, external validity for cognitive performance, the spirometric parameters from the relative perspective showed the greatest diagnostic value of all investigated parameters: Pearson’s bivariate correlations, which were computed for this purpose, were only significant for the increase of VO_2_ (*r*(25) = −0.44, *p* = 0.023) and VCO_2_ (*r*(25) = −0.40, *p* = 0.037) relative to individual baseline values (hypothesis 3a) but not for any of the other investigated absolute/relative parameters (neither EDA nor spirometry).

Further investigating external validity, analyzes of self-reported fear of actually receiving an electric shock during ToS, revealed that fear of shock was correlated significantly with both VO_2_ (*r*(25) = 0.44, *p* = 0.022) and VCO_2_ (*r*(25) = 0.55, *p* = 0.003). Such an association could not be demonstrated for any of the SCL values, nor the relative change measures of VO_2_/VCO_2_ in this study.

Moreover, for discriminant validity (hypothesis 3b) we noted that SCL responsiveness to CBT and ToS was significantly correlated (*r*(23) = 0.70, *p* < 0.001), indicating a lack of discrimination between cognitive load and emotional stress from the relative perspective. Looking at the responsiveness for respiratory parameters, however, we found evidence for a superior discriminating ability, with a significant negative correlation between increments in VCO_2_ in response to the different mental conditions (*baseline-ToS* with *baseline-CBT*, *r*(25) = −0.38, *p* = 0.048).

### Additional analyzes

To gain more in-depth insight into the specificity and sensitivity of the methods studied, ROC analyzes were conducted. Additionally, phasic and tonic portions of the EDA-signal were considered to broaden the picture. For ROC analyzes, area-under-the-curve (AUC) results mirrored those obtained by previously calculated t-tests. In a direct comparison of the AUCs between EDA measures (SCL, tonic, and phasic) and absolute values of VO_2_/VCO_2_, tonic EDA measures initially appear superior in discriminating between baseline and CBT (SCL: 0.485; tonic: 0.859; phasic: 0.685; VO_2_: 0.778; VCO_2_: 0.653) as well as baseline and ToS (SCL: 0.589; tonic: 0.866; phasic: 0.575; VO_2_: 0.637; VCO_2_: 0.590). However, when taking the relative perspective, VO_2_/VCO_2_ show a larger AUC in terms of discriminating a shift from baseline to ToS from a shift from baseline to CBT (SCL: 0.666; tonic: 0.459; phasic: 0.633; VO_2_: 0.797; VCO_2_: 0.701).

## Discussion

In the present study, we used respiratory gases and electrodermal activity in an effort to objectively distinguish different demands (cognitive vs. emotional) in information processing. While EDA has been used extensively in research regarding emotional processing^1^ and gas parameters have mostly been used in sport sciences and medicine^2^, the combined use with respect to cognitive processing is innovative. Even in the context of this relatively simple feasibility study, basic spirometry measures were able to perform equally well or better compared to the diagnostic values of basic (SCL) and more sophisticated EDA measures (basic and tonic portions), as established and refined psychophysiological procedures. Spirometry in psychophysiological application could potentially benefit from more tailored data preparation methods for this purpose as well.

Nevertheless, our results indicate that respiratory gases are promising candidates for the detection and discrimination of different psychological demands. They also exhibit useful and arguably superior specificity and validity (external and discriminant) when compared to established psycho-physiological parameters, namely EDA.

In line with existing research^22^, we demonstrated that EDA is capable of detecting emotional stress. With respect to cognitive load, however, our data indicate that EDA measures are mostly an indicator of the absence of emotional stress. Our results support the notion that respiratory gas parameters can enhance the detection of cognitive load and its discrimination from emotional stress (Fig. 1).

Specifically, VO_2_ and VCO_2_ were sensitive to changes in cognitive load (absolute and relative to baseline), whereas EDA measures were more sensitive to emotional stress than cognitive load. The comparison of gas parameters across both experimental conditions (cognitive load and emotional stress) further supported the specificity of VO_2_, differing significantly between emotional stress and cognitive load. However, we could not find significant evidence for the hypothetical maladaptive respiratory response to emotional stress as proposed by Grassmann et al.^3^ Potentially, a more elaborate approach, rather than simply using the quotient of VCO_2_ and VO_2_ (RER), is required to capture such an effect. Alternatively, a reduced discrimination capability of VCO_2_ is based on greater susceptibility of this parameter to other (non-cognitive) influences^2^.

Concerning discriminant validity, there was a strong intercorrelation of EDA responsiveness for cognitive load and emotional stress induction whereas the lack of such intercorrelation for gas parameters indicated their benefit beyond EDA. In addition, respiratory gas parameters showed superior external validity over EDA, as apparent in significant correlations with both non-physiological parameters (cognitive performance and self-reported fear of shock). In detail, cognitive performance outcomes were negatively related to VO_2_ and VCO_2_ responsiveness. As energy metabolism is arguably a primary mechanism behind variance in gas parameters^8^, these results are in line with previous findings on interrelations of cognitive performance and responsiveness to glucose supplementation^10^. The latter revealed a correlation between performance deficits and the degree of dependence of performance on the consumption of glucose. We suggest that the simultaneous investigation of respiratory gases under cognitive load with differing amounts of glucose supplementation and measures is a promising next step.

## Conclusion

In summary, our experiments revealed different strengths and weaknesses of EDA and spirometry measures, which were most apparent (1) in terms of discriminating baseline activity from each of the two experimental conditions, and (2) with respect to external validity (fear of electric shock during ToS and cognitive performance during CBT). Thus, we think that the combination of spirometry and EDA indeed has added diagnostic value in the detection and discrimination of cognitive load and emotional stress.

Therefore, the study presented here provides an argument for further research into the analysis of respiratory gases in the context of psychological research. In doing so, it demonstrates partly superior, but primarily complementary strengths compared with the established psychophysiological EDA measures. Due to the relatively larger increases in VO_2_ and VCO_2_ compared to that of EDA parameters caused by cognitive load and the opposite relationship under emotional stress, future attempts could be made to identify the different conditions based on the aforementioned and more sophisticated parameters.

Taking into account that we also found changes in respiratory rate, one could argue that this mediates our findings regarding the sensitivity of gas parameters. However, this does not affect the basic ability of spirometry to discriminate episodes of cognitive vs. emotional load. The benefit of this novel use of spirometry is likely limited to physically resting individuals, as metabolic effort from physical activity is likely to override that from mental effort. Conversely, even when dealing with spirometry in the context of non-psychological research, attention should be paid to the possible variance elicited by psychological factors, as demonstrated in the presented experiment. In the present study the focus was to differentiate periods of cognitive load and emotional stress rather than direct stimuli related responses. For future studies it would also be relevant to study spirometry as a psychophysiological method in an experiment with an event-related design^23^.

From a practical point of view, spirometry is still limited by the usually large size of measuring devices and the associated restriction of mobility. A potential solution could be provided by measurement of gas concentrations in the subjects’ periphery or portable devices. We hope that our presented work will spark interest in furthering the use of physiological measures, including spirometry, to obtain objective measures of mental processes.

### Ethical approval

All procedures were approved by the university’s internal review board ("Ethik-Kommission der Bergischen Universität Wuppertal”, Ref.No.: MS/BBL 191,119 Neukirchen).

## Data Availability

The datasets generated during and/or analyzed during the current study are available from the corresponding author on reasonable request.
